# Effective combination chemotherapy with paclitaxel and cisplatin with or without human granulocyte colony-stimulating factor and/or erythropoietin in patients with advanced gastric cancer

**DOI:** 10.1038/sj.bjc.6600345

**Published:** 2002-06-17

**Authors:** G V Kornek, M Raderer, B Schüll, W Fiebiger, C Gedlicka, A Lenauer, D Depisch, B Schneeweiss, F Lang, W Scheithauer

**Affiliations:** Department of Internal Medicine I, University Vienna, Waehringer Guertel 18-20, A-1090 Vienna, Austria; Department of Surgery, General Hospital of Wiener Neustadt, A-2700 Wiener Neustadt, Corvinusring 3-5, Austria; Department of Internal Medicine, General Hospital of Kirchdorf/Krems, A-4560 Kirchdof/Krems, Austria; Department of Surgery, General Hospital of Neunkirchen, A-2620 Neunkirchen, Peischinger Str 19, Austria

**Keywords:** palliative chemotherapy, paclitaxel, cisplatin, human granulocyte colony-stimulating factor, erythropoietin, gastric cancer

## Abstract

A phase II trial was performed to determine the antitumour efficacy and tolerance of combined paclitaxel and cisplatin with or without hematopoetic growth factor support in patients with advanced gastric cancer. Forty-five patients with histologically confirmed metastatic gastric cancer were entered in this trial. Treatment consisted of 2-weekly courses of paclitaxel 160 mg per m^2^ and cisplatin 60 mg per m^2^ both given on day 1. Depending on absolute neutrophil counts on the days of scheduled chemotherapeutic drug administration (1000–2000 per μl), a 5-day course of human granulocyte colony-stimulating factor 5 μg kg^−1^ per day was given subcutaneously; in addition, if haemoglobin was <12.0 mg dl^−1^, erythropoietin 10 000 IU was administered subcutaneously three times per week. The confirmed overall response rate (intent-to-treat) was 44%, including five complete (11%) and 15 partial remissions (33%). Twelve patients had stable disease (27%), 11 (24%) progressed while on chemotherapy, and two patients were not evaluable. The median time to response was 3 months, the median time to progression 7.0 months, and the median survival time was 11.2 months with 12 patients currently alive. Haematologic toxicity was common, though WHO grade 4 neutropenia occurred in only five patients (11%). Apart from total alopecia in 16 patients (36%), severe non-haematologic adverse reactions included grade 3 peripheral neuropathy in six (13%) and anaphylaxis in two patients. In addition, there was one patient each who experienced grade 3 emesis, diarrhea, and infection, respectively. Our data suggest that the combination of paclitaxel and cisplatin with or without G-CSF and/or erythropoietin has promising therapeutic activity in patients with advanced gastric cancer.

*British Journal of Cancer* (2002) **86**, 1858–1863. doi:10.1038/sj.bjc.6600345 www.bjcancer.com

© 2002 Cancer Research UK

## 

Despite its declining incidence in the Western world, gastric cancer is still amongst the most common malignancies (Landis *et al*, 1998). Cytotoxic chemotherapy has been widely used in patients with advanced or metastatic gastric cancer and has been demonstrated to be effective in the palliative management of this disease. In randomised trials, in fact, a significant improvement in overall survival and in quality of life was noted when compared to best supportive care alone (Pyrhonen *et al*, 1995; Scheithauer *et al*, 1995; Glimelius *et al*, 1997). Although several second generation combination regimens that have been developed in the 1980s–including epirubicin/adriamycin/cispatin (EAP), fluorouracil/adriamycin/methotrexate (FAMTX), etoposide/leukovorin/ fluorouracil (ELF), and infusional fluorouracil/cisplatin (FUP)–were reported to result in rather high objective response rates, randomised trials have generally failed to reproduce these data and thus demonstrate a substantial change in the natural course of advanced disease (Wils, 1996; Fuchs, 1997). Only the ECF-regimen (epirubicin, cisplatin and infusional fluorouracil) showed a superior response rate (46% *vs* 21%) and prolonged survival (8.9 *vs* 5.7 months) compared with FAMTX (Webb *et al*, 1997), and thus made it a sort of standard chemotherapy. Still, the identification of new agents and/or drug combinations with a superior therapeutic index remains a principal goal of investigational efforts. Because chemotherapy use in patients with disseminated disease, who fare particularly poorly, is aimed at producing palliative effects, the anticancer activity and side effects must be weighted carefully. The results of recently published trials in patients with metastatic gastric carcinomas have suggested that both cisplatin and paclitaxel are relatively active and well tolerated drugs. Cisplatin has been used in the treatment of gastric cancer in the neoadjuvant, adjuvant and palliative setting and various combination regimens have been reported to show response rates between 20–71% (Findlay *et al*, 1994; Wils, 1996; Morant, 2001). Paclitaxel is a novel antineoplastic drug with the unique cytotoxic mechanism of tubulin stabilisation and polymerisation (Arbuck, 1994). It has demonstrated broad clinical activity in a variety of malignancies both alone and in combination regimens. Antitumour activity of paclitaxel also has been shown in gastric cancer cell lines and in several phase I/II trials (Arbuck, 1994; Bokemeyer *et al*, 1997; Murad *et al*, 1999; Kollmannsberger *et al*, 2000; Safran *et al*, 2000). Paclitaxel is usually well tolerated with myelosuppression being the dose-limiting toxicity and patients receiving this agent can be treated on an outpatient basis.

Favourable results were obtained when both drugs were combined in patients with ovarian cancer, advanced non-small cell lung cancer (NSCLC), head and neck- and oesophageal carcinomas (Ilson *et al*, 1998; Khuri *et al*, 2000; Gatzemeier *et al*, 2000; Pfisterer, 2000). Furthermore, two recently published phase II studies, investigating the combination of cisplatin with docetaxel, a semisynthetic taxoid, suggested a high antitumour activity with an overall response rate of 37 and 56%, respectively (Roth *et al*, 2000; Ridwelski *et al*, 2001).

The aim of the present study was thus to evaluate the antitumour activity and tolerance of paclitaxel plus cisplatin in chemotherapy-naive patients with disseminated gastric cancer. To minimise acute toxicities and counteract myelosuppression that was likely to constitute the dose-limiting toxicity with this combination, we decided to use a biweekly administration schedule of paclitaxel and cisplatin. A potentially improved therapeutic index despite dose intenification by using fractionated, i.e., weekly or biweekly drug dose regimens has been reported in other malignancies, including NSCLC (Brunn, 1997; Soerensen *et al*, 1997). The particular dose regimen used in this trial was based on the results of a previous phase I study in patients with advanced oesophageal cancer (Van der Gaast *et al*, 1997). In addition, to maintain the planned dose intensity, granulocyte colony-stimulating factor (G-CSF) was given depending on absolute neutrophil counts on the days of scheduled chemotherapeutic drug administration. Since recent data suggest that erythropoietin, apart from its ability to potentiate the effect of G-CSF, significantly improves quality of life in cancer patients receiving cisplatin and non-platinum chemotherapy with a possible beneficial effect in terms on overall survival, we decided to co-administer this haematopoetic growth factor in patients with haemoglobin levels below 12 mg μL^−1^ (Pierelli *et al*, 1999; Littlewood, 2001).

## PATIENTS AND METHODS

### Patients selection

Patients eligible for this study had histologically confirmed advanced gastric cancer (except carcinomas of the oesophagastric junction) with bidimensionally measurable disease not previously treated with palliative chemotherapy and not amenable to curative resection. All patients were required to be 75 years old or younger, to have a World Health Organisation (WHO) performance status of less than 3, to have an expected survival time of more than 12 weeks, and to have adequate bone marrow (absolute neutrophil count (ANC) ⩾2.000 per μL, and platelet count ⩾100 000 per μL), adequate renal (serum creatinine concentration <132 μmol, creatinine clearance ⩾70 ml min^−1^ as calculated by the Calvert formula), and adequate hepatic function (bilirubin and serum transaminase level <2 times the upper limit of normal). Patients were ineligible if they had a history of prior or concomitant malignancy, except for curatively treated nonmelanoma skin cancer or *in situ* cervical cancer. Female patients could not be pregnant or lactating. A prior history of atrial or ventricular arrhythmias and/or history of congestive heart failure, even when medically controlled, disqualified patients from study entry. Pre-existing motor or sensory neurologic symptoms ⩾WHO grade 2 were not allowed, nor were active infections or other serious underlying medical conditions that would impair the ability of the patient to receive protocol treatment. All patients gave written informed consent according to institutional regulations.

### Treatment protocol

Chemotherapy consisted of paclitaxel (Taxol®, Bristol-Myers Squibb Company, Princeton, NJ, USA) 160 mg per m^2^, dissolved in 500 ml of normal saline and given as a 3 h infusion plus cisplatin 60 mg per m^2^, diluted in 500 ml normal saline and administered as 1 h infusion. Pre- and post-hydration with at least 2000 ml of intravenous fluid (normal saline or 5% dextrose per 24 h) were mandatory. The recommended prophylactic anti-emetic medication consisted of dexamethasone and 5-hydroxytryptamine-3 antagonists. Before paclitaxel administration, each patient received premedication including dexamethasone (20 mg orally, 12 and 6 h before paclitaxel), cimetidine (300 mg i.v., 30 min before paclitaxel), and diphenhydramine (50 mg i.v., 30 min before paclitaxel). Treatment courses were repeated every 2 weeks, and were to be continued in patients achieving complete or partial remission or stabilisation of disease for a total of 12 courses. Depending on absolute neutrophil counts on the days of scheduled chemotherapeutic drug administration (1000–2000 per μL), a 5-day course of human granulocyte colony-stimulating factor (G-CSF, Neupogen®, Amgen Europe, Netherlands) 5 μg kg^−1^ per day was given. In addition, if haemoglobin was <12.0 mg dl^−1^ erythropoietin 10 000 IU (ERYPO®, Janssen-Cilag Pharma, Austria) was administered s.c. three times per week until a haemoglobin level >13.5 mg dl^−1^ has been reached.

### Toxicity and dosage modification guidelines

Adverse reactions were evaluated according to WHO standard criteria. Treatment could be delayed for up to 2 weeks if the ANC was lower than 1000 per μL and/or platelet count was lower than 75 000 per μL. Prolonged administrations of G-CSF (until the neutrophil count was ⩾2000 per μL) was recommended in the former group of patients. Drug doses were reduced by 25% in case of febrile neutropenia grade 4, if the lowest platelet count was less than 25 000 per μL, in case of grade 1 nephrotoxicity or grade 2 neuropathy, or if any severe (>WHO grade 2) non-haematologic toxicity was observed in the previous cycle. Paclitaxel was stopped in any case of grade 4 skin toxicity and grade 3 anaphylactic reaction. Both chemotherapeutic drugs were discontinued in the event of transitory ⩾ grade 2 renal toxicity or definitive decrease of creatinine clearance (<60 ml min^−1^), ⩾ grade 3 neuropathy, and if severe toxicity recurred despite dose attenuation. Similarly, any patient who required more than 2 weeks for full recovery of adverse reactions (except alopecia and mild neuropathy) was taken off study.

### Pretreatment and follow-up evaluation

Pretreatment evaluation included a complete medical history and physical examination, routine haematology and biochemistry analyses, ECG, chest X-ray, and CT scans to define the extent of disease. Complete blood counts and differential counts were obtained weekly, and biochemical profiles were assessed before each treatment cycle. Measurable lesions were reassessed every 8 weeks by CT scan, X-ray, or any other technique that allows retrospective and independent evaluation.

### Assessment of response

The primary efficacy endpoint of this trial was objective response rate, which was evaluated according to WHO standard criteria. In addition, complete remission (CR) of the primary tumour site was defined as a normal appearing stomach on CT-scan with complete resolution of the endoscopically visible tumour and a negative biopsy of the original site of the tumour. All tumour measurements were reviewed and confirmed by an independent panel of radiologists and oncologists. Secondary efficacy endpoints included the duration of response (measured from the onset of the best response to the date of disease progression), time to progression (calculated from the start of therapy to the time of progression or relapse) and overall survival.

### Statistical methods

An ‘optimal two-stage design’ was chosen for this phase II trial (Gehan, 1961). If fewer than four responses were noted in the first 17 eligible patients, accrual would be halted. Because responses were observed, additional patients were enrolled to a final accrual of 45 patients to better estimate efficacy and characterise the toxicity profile. This sample size was considered sufficient to estimate 95% confidence intervals for the true response rate with a maximum width of 30%. For the response rate, 95% confidence intervals were calculated as previously described (Anderson *et al*, 1985). The distribution of time to death from the time of study entry was estimated by using the Kaplan–Meier product-limit method (Kaplan and Meier, 1958). All patients who were enrolled onto the study were included in the intent-to-treat analysis.

## RESULTS

### Patient characteristics

Between January 1999 and November 2000, a total of 45 patients took part in this trial. All of them were evaluable for toxicity and 43 patients for response assessment. Two patients who were considered non-measurable by the external review committee, were kept in the final intent-to-treat analysis of response. The demographic data, sites of metastatic tumour, and previous therapies are listed in Table 1

**Table 1:**
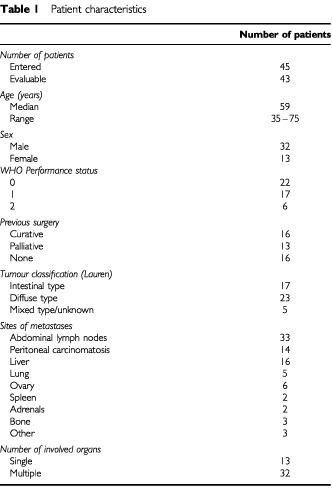
Patient characteristics

. The median age of the 13 female and 32 male patients was 59 years (range, 35 to 75 years), and the median WHO performance status was 1 (range, 0–2). Except for 13 patients, all had multiple metastases involving two or more organ systems. Twenty-nine patients (64%) had disseminated disease at the time of first diagnosis, 13 of whom required palliative surgical interventions. The remaining 16 patients had metastatic disease recurrence after having undergone previous potential curative resection; the median interval from initial diagnosis to relapse was 11 (range, 3 to 131) months in these patients.

A total of 398 courses of study treatment were administered to the 45 patients. The median number of treatment cycles was 10 (range, 2 to 12), and the median duration of follow-up at the time of this analysis was 17 (range, 6–29) months.

### Response to treatment

The overall response rate was 44.4% (95% confidence interval, 29.7 to 60.0%), including five (11.1%) complete and 15 (33.3%) partial remissions; the median duration of response was 6.5 months (range, 3 to 17 months). Twelve additional patients (26.6%) had disease stabilisation, and 11 (24.5%) progressed while on treatment. The median time to disease progression was 7 months (range, 1 to 20) and the median survival was 11.2 months (range, 1.5 to 21+) with 12 patients (26.7%) still being alive after a median follow-up time of 14 months. Sixteen patients, who failed paclitaxel/cisplatin or experienced tumour progression within 6 months after completion of chemotherapy, received second-line therapy. The most frequently used second-line regimens were fluorouracil/leucovorin plus etoposide (*n*=4) or epirubicin (*n*=5), and oxaliplatin/raltitrexed in seven patients.

### Toxicity

Side effects associated with treatment are listed in Tables 2

**Table 2:**
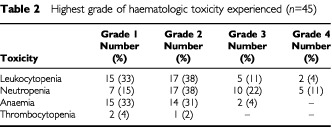
Highest grade of haematologic toxicity experienced (*n*=45)

and 3

**Table 3:**
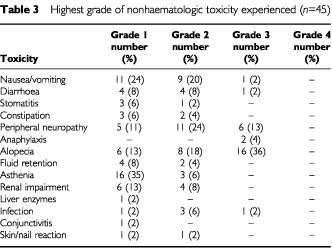
Highest grade of nonhaematologic toxicity experienced (*n*=45)

. Myelosuppression was the most commonly encountered toxicity, although according to the ANC-adapted use of G-CSF, the time to WBC/ANC recovery was generally short: 95% of the episodes of leukopenia/neutropenia were resolved within 7 days. Administration of G-CSF because of ANCs of 1000 to 2000 per μL on the day of scheduled chemotherapeutic drug administration, as indicated in the protocol, was effected in 26 patients (58%), after a median of 6 courses (range, 1 to 12). A total of 59 5-day courses of G-CSF were given with most of the patients (73%) receiving fewer than three courses. Leukocytopenia occurred in 39 patients (87%), and was grade 3 or 4 in five (11%) and two (4%) patients. Neutropenia was also observed in 39 cases (87%), and was grade 3 or 4 in 10 (22%) and five patients (11%), respectively. Five patients (11%) developed documented infection, one of whom required hospitalisation for intravenous antibiotics. Anaemia was commonly observed (69%) and was grade 3 in two patients (4%). Twenty-two patients (49%) received erythropoietin because their pretreatment haemoglobin value was or dropped below 12.0 mg dl^−1^ during chemotherapy and 17 of them (77%) responded to this haematopoetic growth factor support. Grade 1 or 2 thrombocytopenia was seen in only three patients.

Nonhaematologic adverse reactions are listed in Table 3. Gastrointestinal symptoms were the most frequently encountered toxicities. Nausea and vomiting occurred in 21 patients (47%), symptoms however, were generally mild or moderate (grade 1, 2 or 3 in 24, 20 or 2%, respectively) and responsive to standard antiemetic therapy. Diarrhoea was noted in nine patients (20%) and was grade 3 in only one case. Constipation occurred in five patients, and grade 1 or 2 stomatitis in four. Twenty-two patients (49%) developed peripheral neuropathy, including 6 (13%) who experienced severe (WHO grade 3) symptoms. Two patients (4%) developed anaphylactic reactions despite adequate premedication. Skin reactions and nail changes were seen in two patients each, and minor fluid retention in six. Alopecia was noted in a total of 30 patients (67%), 16 of who had complete hair loss. Asthenia was reported by 19 (42%) patients. Transiently impaired liver and renal function were observed in one (2%) and 10 (22%) patients, respectively.

### Drug exposure

The dose of both chemotherapeutic drugs was reduced in three patients due to (febrile) grade 4 neutropenia and in two patients because of (grade 1) nephrotocixity; dose modifications of paclitaxel according to progressive peripheral neuropathy were required in five additional patients. Fifteen patients (33%) had at least one treatment delay of 1 week at some time during therapy, and the total number of delayed courses was 29 (14%). The reasons for delayed courses were haematologic toxicity in 20 cases, neutropenic fever in four, transiently impaired renal function in one, deterioration in performance status in two, and personal reasons in two patients. Treatment was discontinuated prematurely in two patients because of anaphylaxis, in four patients due to grade 3 peripheral neuropathy (after six to 10 treatment courses), in one case because of intercurrent upper gastrointestinal tract bleeding, and in two additional patients for personal reasons.

Dose-intensity was calculated for each patient and for each drug. The mean given dose-intensity of the combination was 92% of the projected dose. The mean delivered dose of paclitaxel per week was 73.6 mg per m^2^ (range, 59.2 to 80 mg per m^2^) and the mean delivered dose of cisplatin per week was 27.6 mg per m^2^ (range, 22.2 to 30 mg per m^2^).

## DISCUSSION

The continuing lack of substantial progress in the treatment of advanced gastric cancer, particularly in patients with poor performance status or compromised organ function, who are unlikely to tolerate potentially active but toxic regimens, has prompted investigators to evaluate new agents and/or drug combinations including docetaxel, paclitaxel, and irinotecan (Bokemeyer *et al*, 1997; Boku *et al*, 1999; Murad *et al*, 1999; Kollmannsberger *et al*, 2000; Roth *et al*, 2000; Ridwelski *et al*, 2001).

The present study, to our knowledge, is the first to report mature results on the efficacy and safety with the combination of paclitaxel plus cisplatin in patients with advanced gastric cancer. The rationale for their combined use were: (1) the documented activity of both drugs in gastric cancer when used as single agent (Wils, 1996); (2) the apparent synergistic efficacy and safety of this combination in patients with ovarian-, head and neck cancer, and NSCLC (Ilson *et al*, 1998; Gatzemeier *et al*, 2000; Khuri *et al*, 2000; Pfisterer, 2000); (3) as well as the encouraging results of two recently published reports of a combination of cisplatin and the semisynthetic taxoid docetaxel (Roth *et al*, 2000; Ridwelski *et al*, 2001).

With an intent-to treat response rate of 44%, including 11% complete remissions, a median progression free interval of 7 months, and median overall survival of 11.2 months, the results of the present multicenter phase II investigation suggest a marked antitumour activity of this combination in patients with metastatic gastric cancer. The non-randomised phase II study design, as we have learned in the past, does not allow to draw any firm conclusion nor any direct comparison with other regimens. In fact, other drug combinations with even higher objective response rates than observed in the present study have been described in the past, and have failed to show superiority in subsequent phase III trials (Kelsen *et al*, 1998; Vanhoefer *et al*, 2000). Therefore, results must be interpreted with caution, and warrant confirmation in a randomised trial setting. Still, the observed antitumour potential, which is in agreement with the previously mentioned phase II studies of Roth *et al* (2000) (RR 56%, median survival 9 months), and Ridwelski *et al* (2001) (RR 37%, median survival 10.4 months), suggests that taxane/cisplatin-based combination chemotherapy might be as active as second- or even third-generation regimens including ECF or the more intense and toxic PELF (Cascinu *et al*, 1997; Webb *et al*, 1997). Potential advantages of the described biweekly and other taxane+cisplatin combination regimens are related to the non-requirement of a central venous access and external infusional devices with their associated risks and costs (Lemmers *et al*, 1996; Kock *et al*, 1998).

As it concerns the tolerance of treatment, neutropenia was a commonly encountered adverse reaction associated with this regimen, and WHO grade 3 or 4 toxicity occurred in 15 patients (33%). Due to the actual ANC-adapted use of G-CSF, however, only 20 of 398 courses (5%) had to be delayed for haematologic toxicity reasons, and there was a low rate of febrile neutropenic episodes with only one patient requiring hospitalisation. The rather high rate/incidence of anaemia in our patient population was probably not only caused by therapy, but also influenced by the underlying malignant disease. Twenty-two patients received recombinant human erythropoietin according to the protocol and 17 of them (77%) responded to the haematopoetic growth factor support. In as much reversing anaemia in these patients has contributed to the favourable study outcome can not be determined. Clinical trials, however, have shown that correcting anaemia not only provides an objective improvement in the patients' well being, but may also improve response and possibly the duration of survival (Littlewood, 2001; Littlewood *et al*, 2001).

Apart from anaphylactic reactions in two patients and grade 3 peripheral neurotoxicity in six, nonhaematologic adverse reactions were generally mild to moderate and fully reversible. Despite use/realisation of a high dose intensity of both chemotherapeutic drugs, in addition to a lower rate of neutropenia, also certain nonhaematologic toxicities such as arthralgia/myalgia, asthenia and gastrointestinal symptoms were less commonly seen when compared to other phase II/III studies of this particular drug combination in other disease entities (McGuire *et al*, 1996; Rose *et al*, 1999; Gatzemeier *et al*, 2000). Similarly, in the advanced gastric cancer trials of Roth *et al* (2000) and Ridwelski *et al* (2001), investigating docetaxel and cisplatin at a dose of 75–85 mg per m^2^ once every 3 weeks, a much higher rate of severe haematotoxicity (up to 57% of patients experienced grade 4 neutropenia), and grade 3 gastrointestinal adverse reactions (which occurred in about 9% in both trials) was noted.

In conclusion, the results of this trial indicate that the described biweekly combination regimen of paclitaxel plus cisplatin±G-CSF and/or erythropoietin is an effective and tolerable regimen for disseminated gastric cancer. It seems to have durable antitumour activity with an acceptable level of both haematologic and other organ toxicities. Compared with previous phase II/III investigations of this combination in other malignancies, the incidence of severe neutropenia as well as of certain other nonhaematologic adverse reactions seems to be lower. The difference might be explained by the ANC-adapted use of G-CSF±erythropoietin, the choice of the taxane, and/or the biweekly chemotherapeutic drug administration schedule. In view of the promising therapeutic index of the described regimen, further evaluation of this combination in patients with advanced disease as well as in the neoadjuvant setting, seems warranted. In these and/or other clinical trials in patients with gastric cancer receiving cytotoxic chemotherapy, it might also be of particular interest to try to define precisely the impact of correcting anaemia with erythropoietin in terms of quality of life and therapeutic outcome.

## ACKNOWLEDGEMENTS

Supported in part by the Gesellschaft zur Erforschung der Biologie und Behandlung von Tumorkrankheiten.
